# Simple Immunosensor Based on Carboxyl-Functionalized Multi-Walled Carbon Nanotubes @ Antimony-Doped Tin Oxide Composite Membrane for Aflatoxin B_1_ Detection

**DOI:** 10.3390/mi14050996

**Published:** 2023-05-03

**Authors:** Guanglei Chu, Zengning Liu, Yanyan Zhang, Yemin Guo, Xia Sun, Ming Li

**Affiliations:** 1 School of Agriculture Engineering and Food Science, Shandong University of Technology, No. 12 Zhangzhou Road, Zibo 255049, China; chuguanglei1994@163.com (G.C.); gym@sdut.edu.cn (Y.G.); sunxia2151@sina.com (X.S.); 2 Hunan Agricultural Equipment Research Institute, Hunan Academy of Agricultural Sciences, No. 120 Donghu Road, Changsha 410125, China; 3 Shandong Provincial Engineering Research Center of Vegetable Safety and Quality Traceability, No. 12 Zhangzhou Road, Zibo 255049, China; liuzengning@163.com; 4 College of Design and Engineering, National University of Singapore, No. 21 Lower Kent Ridge Road, Singapore 119077, Singapore

**Keywords:** immunosensor, aflatoxin B_1_, multi-walled carbon nanotubes, antimony-doped tin oxide, field real-time detection

## Abstract

This paper presents a novel nano-material composite membrane for detecting aflatoxin B_1_ (AFB_1_). The membrane is based on carboxyl-functionalized multi-walled carbon nanotubes (MWCNTs-COOH) @ antimony-doped tin oxide (ATO)-chitosan (CS). To prepare the immunosensor, MWCNTs-COOH were dissolved in the CS solution, but some MWCNTs-COOH formed aggregates due to the intertwining of carbon nanotubes, blocking some pores. ATO was added to the solution containing MWCNTs-COOH, and the gaps were filled by adsorbing hydroxide radicals to form a more uniform film. This greatly increased the specific surface area of the formed film, resulting in a nano-composite film that was modified on screen-printed electrodes (SPCEs). The immunosensor was then constructed by immobilizing anti-AFB_1_ antibodies (Ab) and bovine serum albumin (BSA) on an SPCE successively. The assembly process and effect of the immunosensor were characterized using scanning electron microscopy (SEM), differential pulse voltammetry (DPV), and cyclic voltammetry (CV). Under optimized conditions, the prepared immunosensor exhibited a low detection limit of 0.033 ng/mL with a linear range of 1 × 10^−3^–1 × 10^3^ ng/mL. The immunosensor demonstrated good selectivity, reproducibility, and stability. In summary, the results suggest that the MWCNTs-COOH@ATO-CS composite membrane can be used as an effective immunosensor for detecting AFB_1_.

## 1. Introduction

Aflatoxin (AFT), a potent natural carcinogen, is a biologically active secondary metabolite mainly produced by Aspergillus flavus, Aspergillus parasiticus, and Aspergillus oryzae [1]. Among them, Aflatoxin B_1_ (AFB_1_) is the most toxic and widespread, posing a significant threat to human health [2]. It is almost impossible to avoid AFB1 in agricultural products, and its continuous consumption, even at low levels, can lead to a dramatic increase in the incidence of cancer and other diseases, particularly in developing countries, where AFB1 is associated with many types of cancers. Therefore, the maximum residue levels of AFB1 in foods must be strictly controlled. Traditional methods of AFB1 detection are time-consuming and not suitable for on-site monitoring of agricultural products. Hence, there is an urgent need to develop rapid and quantitative methods and instruments for AFB1 detection to achieve effective control of its levels in agricultural products.

To safeguard food safety and protect human health, a range of instrument detection techniques have been developed, such as thin-layer chromatography (TLC) [3], high-performance liquid chromatography (HPLC) [4], and HPLC-mass spectrometry (MS) [5]. Although these methods offer high detection accuracy and stability, their complex and time-consuming operations have hindered their advancement in the realm of rapid detection [6]. Hence, the need of the hour is to devise a simple, swift, and efficient means of detecting AFB1. In recent years, immunosensors have emerged as a promising detection method that leverages the specific binding of antigens and antibodies to detect small molecules [7]. Owing to their high detection accuracy, low cost, time efficiency, and ease of use, a range of immunosensors have been deployed to detect AFTs [8]. Among the electrochemical signal transduction techniques, electrochemiluminescence immunosensors [9] and electrochemical immunosensors [10] have exhibited high sensitivity and are widely applied in diverse immune reactions using voltammetry and amperometry, which are the most prevalent and widely adaptable methods [11]. Enhancing the current response of the immunosensor through the incorporation of nanomaterials represents an effective means of heightening its sensitivity. Nanomaterials such as gold nanoparticles [12], Fe_3_O_4_ [13], and graphene oxide [14] are typically employed to modify electrodes. This incorporation not only improves the transmission efficiency on the surface of the electrode but also amplifies the specific surface area.

Chitosan, a naturally-occurring alkaline polysaccharide compound, is also known as polyglucosamine (1-4)-2-amino-B-D glucose. It is obtained by the deacetylation of chitin, and is highly versatile due to its abundant amino groups. When chitosan is dissolved in acidic solution, these amino groups become free and protonated, giving it polycationic electrolyte properties. This remarkable feature allows chitosan to serve as a highly effective dispersant, capable of chelating various heavy metal ions. In addition, chitosan exhibits exceptional anion adsorption capabilities and is naturally biodegradable, making it a highly attractive and eco-friendly option [15].

Multi-walled carbon nanotubes (MWCNTs) possess remarkable electrical conductivity, thermal conductivity, and mechanical properties, rendering them ideal for developing high-performance polymer composites with multifunctional capabilities. The unique structure and high specific surface area of MWCNTs allow for precise control of electronic properties through molecular adsorption, doping, and charge transfer [16]. Furthermore, the deposition of MWCNTs onto electrode surfaces can create a continuous, ordered arrangement on the electrode, establish a favorable micro-environment for immobilizing enzymes, aptamers, or antibodies, and provide a pathway for electron transfer [17]. However, nano-films of MWCNTs have the tendency to non-specifically adsorb small molecules, which may lead to inaccuracies in small molecule detection.

Nanostructured doped oxides have become a focal point in the realm of research due to their remarkable electrochemical properties [18]. Among them, antimony-doped tin oxide (ATO) stands out as a promising alternative, as it enhances the conductivity of the doping agent without compromising on high optical transparency [19]. Additionally, ATO also exhibits exceptional electrocatalytic performance [20]. When added to a solution of carboxyl-functionalized multi-walled carbon nanotubes (MWCNTs-COOH), ATO engulfs the aggregates of MWCNTs-COOH to generate novel nanocomposites. The integration of ATO results in a denser and more uniform nanocomposite film, which in turn reduces the interaction between MWCNTs-COOH and the reaction substrate, thus lowering the rate of electron transfer.

In this work, taking advantage of the combination of MWCNTs-COOH and ATO nanomaterials, we developed nanocomposite membranes and used them for the construction of the AFB_1_ immunosensor. The aim of this work is to provide a sensitive and stable membrane for quantitative determination of AFB_1_ in food and agricultural products.

## 2. Experimental

### 2.1. Apparatus

The cyclic voltammetry (CV) and differential pulse voltammetry (DPV) measurements were conducted using a CHI660D electrochemical workstation purchased from Shanghai Chenhua Co., Shanghai, China. The screen-printed carbon electrodes (SPCEs, TE100, d = 3 mm) used in the experiment were purchased from Zensor R&D (Taiwan, China). The morphology of the modified electrodes was observed using a scanning electron microscope (SEM) from the Netherlands. Ultrasonication was performed using a SK3300H ultrasonic cleaner from Shanghai, China, and the solution was blended using a PTR-35 SPC vortex mixer from Britain. All experiments were performed at room temperature.

### 2.2. Reagents and Materials

The antibodies (Abs) were procured from the Oil Crops Research Institute of the Chinese Academy of Agricultural Sciences (Wuhan, China). The antimony-doped tin oxide (ATO) with an OD of 20 nm and purity of 99% and carboxyl-functionalized multi-walled carbon nanotubes (MWCNTs-COOH) with an OD range of 10–20 nm were purchased from Beijing Gold Deco Island Co., Ltd. (Beijing, China). Potassium ferrocyanide (K_3_[Fe(CN)_6_]) and potassium ferricyanide (K_4_[Fe(CN)_6_]) were purchased from Yongda Chemical Reagent Co., Ltd. (Tianjin, China). The 0.01 M pH 7.2–7.4 phosphate buffer solutions (PBS) were procured from Beijing Solarbio Science & Technology Co., Ltd., Beijing, China. All other chemicals used were of analytical reagent grade. Ultrapure water (18.2 MΩ·cm) purified with an LS MK_2_ PALL-water purification system was used for the preparation of all solutions. Chitosan (CS) was obtained from Sangon Biotech Co., Ltd. (Shanghai, China).

### 2.3. Preparation of MWCNTs-COOH @ ATO-CS Composites

A total of 0.2 g of CS was dissolved in 100 mL of 1% acetic acid solution, and magnetic stirring was continued for more than 8 h to prepare a 0.1% (*w*/*v*) CS solution. Then, 2 mg of MWCNTs-COOH powder and 1.2 mg of ATO powder were accurately weighed and dissolved in 4 mL of CS solution using 2 h of ultrasonic treatment and 2 h of mixing with a mixer until the suspension became uniform and stable. The resulting highly dispersed and dark grey suspension was the MWCNTs-COOH @ ATO-CS solution, which was stored in a refrigerator (4 °C) for the experiment.

### 2.4. Preparation of Immunosensor Based on SPCEs

#### 2.4.1. Preparation of SPCEs

To prepare the electrode for modification, it was thoroughly cleaned by ultrasonic cleaning in NaOH and HCl solutions for 5 min each. The electrode was then washed with ultrapure water and dried with nitrogen gas. To further ensure cleanliness, the electrode was washed with anhydrous ethanol and dried again with nitrogen gas. The treated electrodes were then immersed in pH 5.0 PBS solution for 300 s with a potential of 1.75 V and subsequently scanned from 0.3 V to 1.25 V and from 0.3 V to −1.3 V until a steady state CV curve was obtained [21].

#### 2.4.2. Preparation of AFB_1_/BSA/Ab/MWCNTs-COOH @ ATO-CS/SPCEs Immunosensor

**Figure 1:**
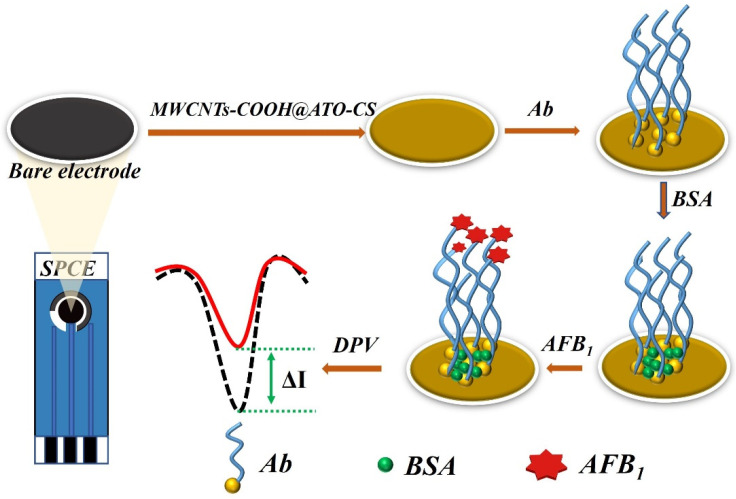
Schematic diagram of the preparation process of the immunosensor.

A total of 6 μL MWCNTs-COOH @ ATO-CS solution was applied to the pre-treated SPCE surface and allowed to dry at room temperature, creating the MWCNTs-COOH @ ATO-CS/SPCE. Next, 6 μL of Ab was added to the modified electrode surface to form Ab/MWCNTs-COOH@ATO-CS/SPCE. To create the BSA/Ab/MWCNTs-COOH@ATO-CS/SPCE, 6 μL of BSA was added to the nanomaterial-modified electrode surface and dried at −4 °C. The electrode was then stored at 4 °C for AFB_1_ detection. The preparation process of the immunosensor is illustrated in Figure 1.

#### 2.4.3. Electrochemical Measurements

The composites of MWCNTs-COOH @ ATO-CS were subjected to scanning electron microscopy (SEM) to analyze their properties. All electrochemical measurements were carried out in 15 mL of 0.1 M PBS (pH 7.0) that contained a 1:1 mixture of 5 mM K_3_[Fe(CN)_6_]/K_4_[Fe(CN)_6_] as a redox probe and 0.1 M KCl at ambient temperature. CV was conducted within a potential range from −1.0 V to 1.0 V at a scan rate of 100 mV/s. Electrochemical differential pulse voltammetry (DPV) measurements were performed under the following conditions: voltage was scanned from −0.30 V to 0.60 V with a pulse height of 100 mV, while the step height and frequency were maintained at 4 mV and 15 Hz, respectively. To investigate the sensitivity and specificity of the proposed immunosensor, DPV was utilized. Additionally, parameters that affected the immunosensor response, such as the Ab concentration and response time, were optimized. Following optimization, the proposed immunosensor was used to detect AFB_1_.

### 2.5. Immunosensor Specificity Analysis

In order to validate the specificity of the immunosensor, control experiments were conducted using five different fungal toxins, namely, AFM_1_, α-zearalenone (α-ZEN), zearalenone (ZEN), ochratoxin A (OTA), and fumonisin B_1_ (FB_1_) at a concentration of 5 μg/mL. In addition, AFB_1_ at a concentration of 100 ng/mL was also detected using the same sensor under unchanged experimental conditions. The detected currents from the control experiments were compared to observe any differences in response.

### 2.6. Peanut Oil Sample Pretreatment Method

Weigh precisely 5 g of peanut oil into a 100 mL triangular flask, and then add 25 mL of 10% methanol PBS solution. Different concentrations of AFB_1_ should be added and the mixture should be shaken vigorously with an oscillator for 30 min to ensure thorough mixing. Next, the mixture should be centrifuged in a centrifuge for 10 min at 10,000 r/min. After centrifugation, 1 mL of the supernatant should be taken and 4 mL of methanol PBS solution should be added. Vigorous shaking for 5 s is necessary to ensure proper mixing, and then the sample should be stored in a refrigerator for further use.

## 3. Results and Discussion

### 3.1. Characterizations of Modified Electrodes

**Figure 2:**
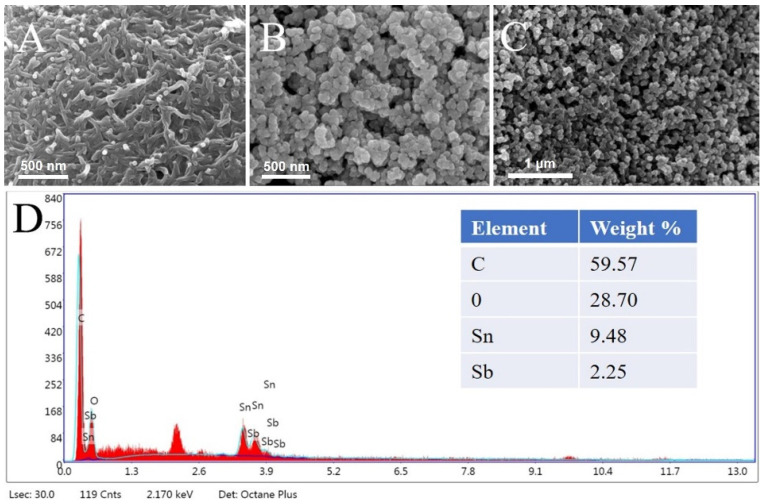
SEM images of (**A**) MWCNTs-COOH-CS, (**B**) ATO-CS, (**C**) MWCNTs-COOH @ ATO-CS, (**D**) EDS image of MWCNTs-COOH @ ATO-CS.

The modified electrodes were characterized using scanning electron microscopy (SEM) to investigate the morphology and structure of the prepared materials. The SEM images in Figure 2A revealed the presence of nano-scale cracks and holes on the electrode surface after the MWCNTs-COOH-CS composite film was modified onto the electrode. Figure 2B showed uniform density and size distribution of ATO-CS nanoparticles. The SEM image in Figure 2C depicted the morphology of MWCNTs-COOH @ ATO-CS. The MWCNTs-COOH formed clusters that were wrapped by a layer of ATO through electrostatic interaction, which increased the specific surface area of the nanocomposite material and made the material distribution more uniform. Compared to MWCNTs-COOH-CS or ATO-CS alone, MWCNTs-COOH @ ATO-CS was denser, which helped to increase the transfer rate of electrons. During the early stage of the formation of MWCNTs-COOH, they were prone to stacking together layer by layer, and many cracks or gaps appeared during the stacking process, resulting in the surface of MWCNTs-COOH being full of small holes. However, the addition of ATO compensated for these gaps and improved the uniformity of the nanocomposite, while also greatly enlarging its specific surface area. The EDS image in Figure 2D showed the analysis of the composition of C, O, Sn, Sb, and other elements in MWCNTs-COOH @ ATO-CS.

**Figure 3:**
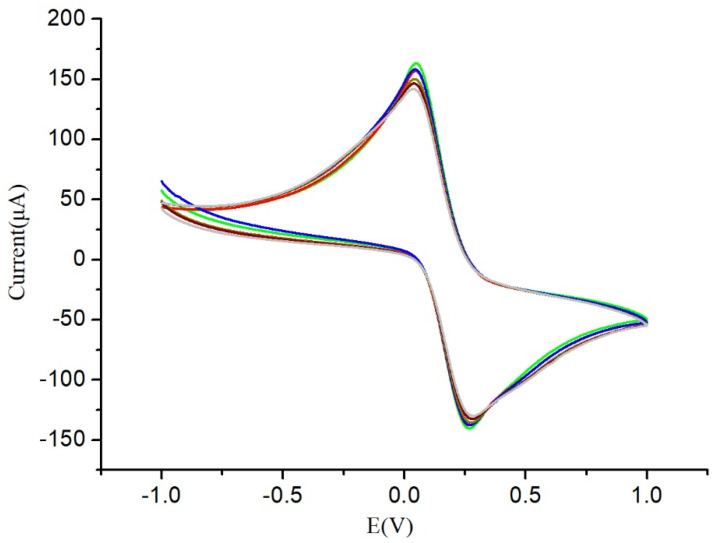
Stability of MWCNTs-COOH @ ATO.

In addition, the stability of the MWCNTs-COOH @ ATO modified electrode was also evaluated. Six consecutive tests were conducted on the modified SPCE using the nanomaterials. As depicted in Figure 3, the six curves exhibited a high degree of consistency, suggesting that the nanocomposites demonstrated excellent stability.

### 3.2. Electrochemical Behavior of the Modified Electrodes

**Figure 4:**
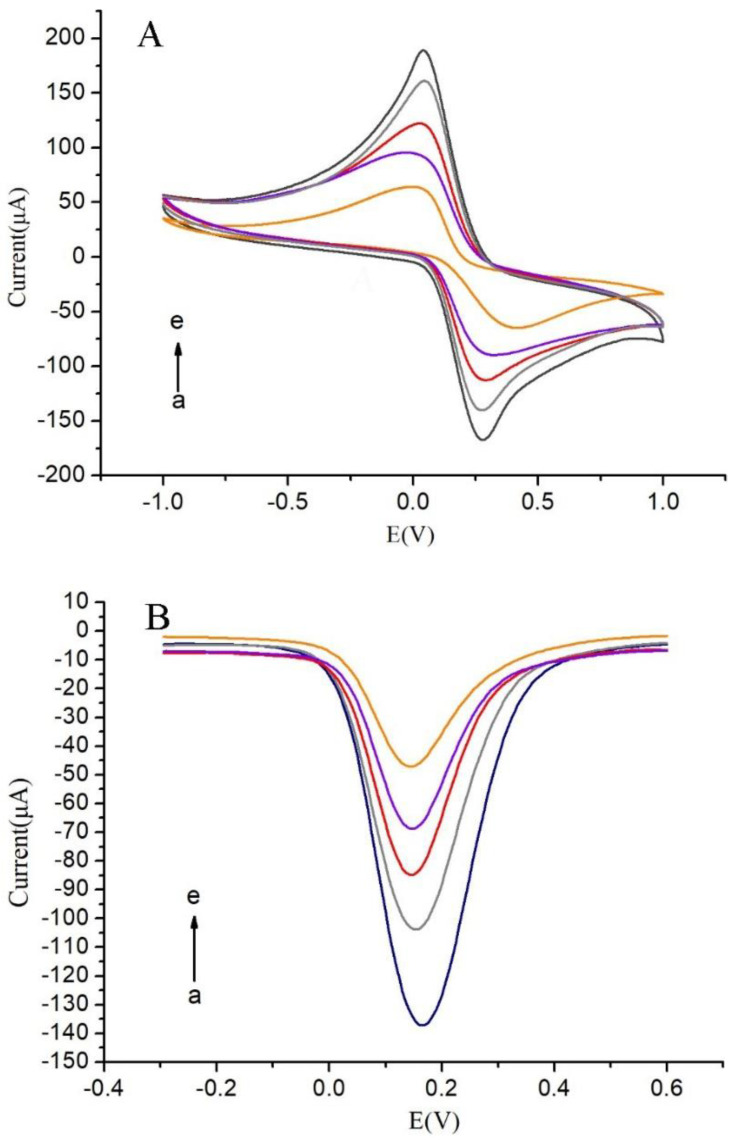
(**A**) CVs of modified screen-printed carbon electrodes; (**B**) DPVs of modified screen-printed carbon electrodes a: MWCNTs-COOH @ ATO-CS/SPCE, b: Ab/MWCNTs-COOH @ ATO-CS/SPCE, c: BSA/Ab/MWCNTs-COOH @ ATO-CS/SPCE, d: SPCE, e: AFB_1_/BSA/Ab/MWCNTs-COOH @ ATO-CS/SPCE.

The assembly process of the immunosensor was investigated using CV and DPV techniques, where the bottom liquid contained 5 mM [Fe(CN)_6_]^3−/4−^ and 0.1 M KCl. As illustrated in Figure 4, the redox peaks of bare SPCEs were evident. Upon modification of the electrode with MWCNTs-COOH @ ATO, the peak current increased substantially to 160 μA (curve b), suggesting that the MWCNTs-COOH @ ATO possessed a large surface area and good electronic conductivity. Since antibodies lack conductivity as macromolecular proteins, their addition impeded electron transfer between [Fe(CN)_6_]^3−/4−^ and the electrode surface. BSA acted as a blocker, eliminating non-specific binding by closing specific sites on the surface of the electrode. Furthermore, since BSA had no conductivity, electron transfer was further obstructed, resulting in a rapid decrease in peak current. These results imply that the antibodies and BSA were successfully immobilized on the electrode surface.

### 3.3. Optimization Parameters of the Immunosensor Performance

**Figure 5:**
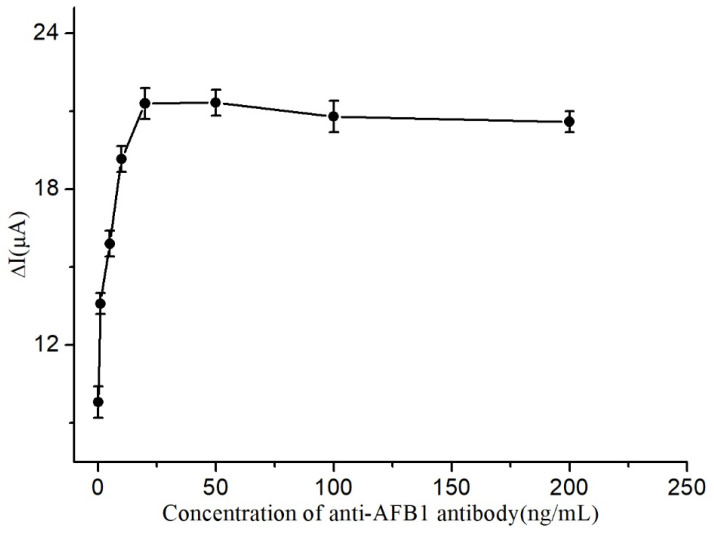
Influence of immunosensor concentration.

The impact of Ab concentration on the immunosensor’s response was investigated and the results are presented in Figure 5. As shown in the figure, the peak current increased gradually with increasing Ab concentration until it reached a maximum value at 20 ng/mL. Subsequently, the response remained relatively stable as the Ab concentration continued to increase, indicating that the maximum number of Ab molecules had already been immobilized on the electrode surface. Thus, 20 ng/mL was identified as the optimal Ab concentration for fabrication of the immunosensor.

**Figure 6:**
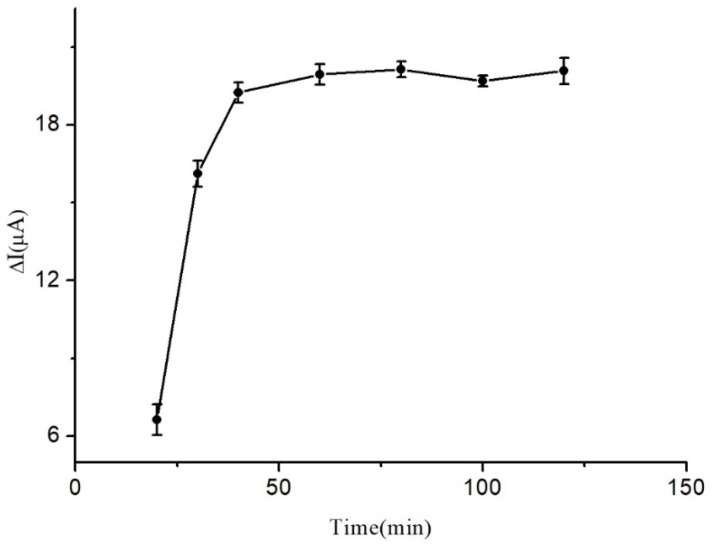
Influence of reaction time.

A study was conducted to investigate the effects of reaction time on the response of the immunosensor, and the findings are illustrated in Figure 6. It was observed that the peak current increased in a gradual manner with the increase in reaction time and reached its highest value at 40 min. Furthermore, the response became almost stable as the reaction time was further extended, indicating that the reaction time had reached a saturation point on the immunosensor. Based on these observations, 40 min was deemed the most suitable reaction time for the fabrication of the immunosensor.

### 3.4. Calibration Curve

The unknown solution’s AFB_1_ concentration was deduced by analyzing the current response of the immunosensor at various AFB_1_ concentrations immobilized on the electrode surface. A correlation between different AFB_1_ concentrations and the immunosensor was established, revealing that as the AFB_1_ concentration increased, the current response difference decreased.

**Figure 7:**
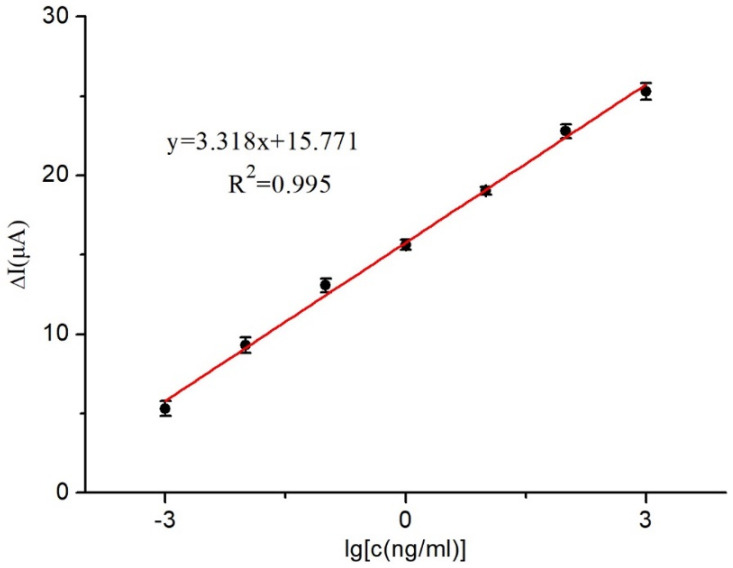
Relationship between current change and AFB_1_ concentrations.

Figure 7 demonstrates a remarkable linear relationship between the current variance and the corresponding AFB_1_ concentration. With the ideal experimental conditions, the AFB_1_ concentration could be accurately determined in the range of 1 × 10^−3^ to 1 × 10^3^ ng/mL using the linear equation y = 15.771 + 3.318x (R^2^ = 0.995). The immunosensor also exhibited a remarkable detection limit of 0.03 ng/mL (S/N = 3).

### 3.5. Selectivity and Stability of Immunosensor

**Figure 8:**
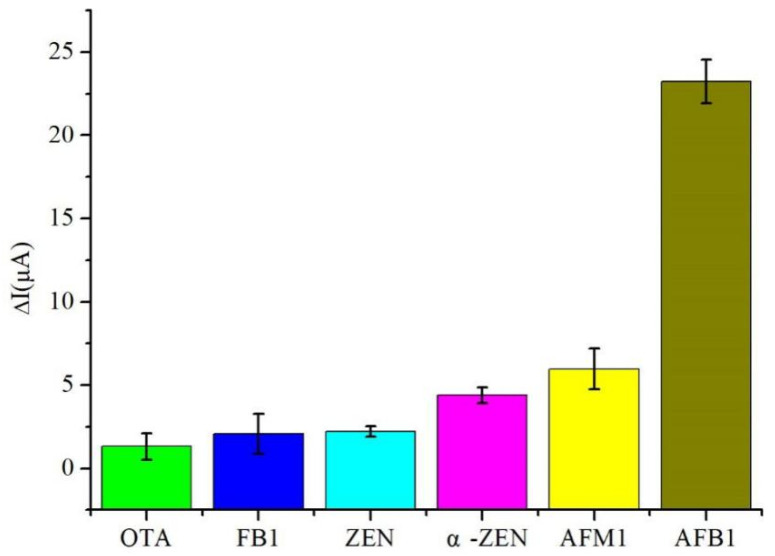
The selectivity for five kinds of mycotoxins (5 μg/mL): α-zearalenone (α-ZEN), ochratoxin A (OTA), fumonisin B_1_ (FB_1_), zearalenone (ZEN) and AFM_1_.

The selectivity of the immunosensor was evaluated by comparing the sensing results of five different mycotoxins: α-zearalenone (α-ZEN), ochratoxin A (OTA), fumonisin B1 (FB_1_), zearalenone (ZEN), and AFM1, all at concentrations of 5 μg/mL, while the concentration of AFB_1_ was maintained at 100 ng/mL. As illustrated in Figure 8, except for AFM_1_, the current response of other toxins was lower than 5 μA. Although AFM_1_ exhibited a current response of 7 μA, its concentration was 50 times higher than that of AFB_1_. These findings indicate that the developed immunosensor possessed an excellent selectivity for practical applications.

To ensure the success of the immunosensor preparation, the stability of the prepared immunosensors was assessed as a crucial factor. Following optimized conditions, a total of 12 immunosensors were fabricated and stored at 4 °C for 15 days. Subsequently, these immunosensors were used to detect AFB_1_ at a concentration of 100 ng/mL. The results revealed a slight decrease of approximately 9.2% in the measured current difference, indicating that the immunosensor exhibited good stability.

### 3.6. Determination of Spiked Recovery of AFB1 in Peanut Oil

To assess the accuracy of the immunosensor, spiked recoveries were measured in peanut oil samples after pretreatment. AFB_1_ was added to the peanut oil samples at spiked concentrations of 10^−1^ ng/mL, 10 ng/mL, and 10^2^ ng/mL. These experiments were conducted under optimal conditions, and the results are presented in Table 1. The results demonstrated spiked recoveries ranging from 95.15 to 111.60%, with relative standard deviations ranging from 2.3 to 5.3%. These findings suggest that the immunosensor exhibited good detection accuracy.

### 3.7. Sensor Performance Comparison

Referring to Table 2, to conduct a comprehensive evaluation of the sensor developed in this study, we compared and assessed various performance indicators of immune sensors. Notably, our sensor boasts a wider detection range and a superior detection limit, which suggests its great potential for practical applications.

## 4. Conclusions

This study aimed to develop an AFB_1_ immunosensor using a nanocomposite material, MWCNTs @ ATO-CS, via an experimental approach. The nanocomposite film was created by incorporating multi-walled carbon nanotubes with nano-stannic oxide antimony and CS as a dispersant, resulting in a film with excellent adhesion and biocompatibility. SEM and CV were used to characterize the nanocomposite film, which exhibited a high level of uniformity, allowing for amplified current signal detection in the immunosensor and facilitating the immobilization of Ab. The immunosensor was constructed by immobilizing MWCNTs @ ATO-CS, Ab, and BSA on SPCE through the layer-wise self-assembly method. Each step of sensor fabrication was characterized using CV and DPV. Parameters, such as the loading of antibodies and the incubation time of the immune reaction, were optimized. The calibration curve was established under optimal conditions, showing a linear relationship for AFB_1_ in the range of 10^−3^ to 10^3^ ng/L with a linear equation of y = 3.318x + 15.771 (R^2^ = 0.995). The immunosensor also demonstrated a limit of detection of 0.03 ng/L (S/N = 3). The specificity and stability of the immunosensor were verified, demonstrating excellent performance. Furthermore, the spiked recovery detection test in peanut oil samples illustrated the immunosensor’s potential for practical sample detection.

## Figures and Tables

**Table 1:** Detection of peanut oil by immunosensor.

Sample	AFB_1_ Addition(ng/mL)	Standard Current Difference (μA)	ΔI (μA)	RSD (%, *n* = 5)	Recovery Rate (%)
1	10^−1^	5.82	5.6	5.2	95.15
2	10	9.14	10.2	3.2	111.60
3	10^2^	12.45	12.08	2.3	96.10

**Table 2:** Comparison of the performance of the sensor prepared in this study to other immunosensors.

Sensors	Detection Method	LOD	Linear Rang	Practical Samples	Ref.
Porous AuNPs/GCE	DPV	0.94 ng/mL	0.01–20 ng/mL	Glutinous rice/Corn/Rice	[22]
Au/Bi_2_S_3_/ERGO/CF	DPV	8 pg/mL	10 pg–20 ng/mL	Cornflour	[23]
MWCNTs/RTIL/Ab/AFB_1_/GCE	EIS	0.03 ng/mL	0.1–10 ng/mL	Olive oils	[24]
BSA/Ab/MWCNTs-COOH @ ATO-CS/SPCEs	DPV	0.033 ng/mL	1 × 10^−3^–1 × 10^3^ ng/mL	Peanut oil	This Work

GCE: glassy carbon electrode, ERGO: electrochemically reduced graphene oxide, CF: carbon fiber, RTIL: room temperature ionic liquid, EIS: electrochemical impedance spectroscopy.

## Data Availability

Not applicable.
